# The effect of pomegranate peel extract added as a natural preservative on the quality parameters of thornback ray (*Raja clavata*) sausages stored at +4°C

**DOI:** 10.1002/fsn3.4207

**Published:** 2024-06-11

**Authors:** Emre Caglak, Ozen Yusuf Ogretmen, Baris Karsli

**Affiliations:** ^1^ Department of Seafood Processing Technology, Faculty of Fisheries Recep Tayyip Erdoğan University Rize Turkey

**Keywords:** fish sausage, natural additive, pomegranate peel extract, refrigerated storage, shelf life, thornback ray

## Abstract

In this study, three different groups of sausages were produced from thornback ray (*Raja clavata*) without additives (control group), waste pomegranate peel extract (natural group), and ascorbic acid (synthetic group). Biochemical, physicochemical, and microbiological changes of sausages were examined under refrigerator conditions (+4°C), and the shelf life was determined. The best results in terms of nutritional and physicochemical values were obtained in sausages treated with pomegranate peel extract. All sausage groups were spoiled on the 15th day in terms of the total volatile basic nitrogen (TVB‐N); however, the pomegranate peel extract group showed a more positive effect compared to the other sausage groups. However, this value was not considered because cartilaginous fish such as stingrays contain higher levels of nonprotein nitrogenous compounds. It was observed that microbial growth was less in the natural group and the antimicrobial effect of pomegranate peel extract was higher than that of ascorbic acid. In addition, it was determined that the pomegranate peel extract group extended the shelf life up to 6 days in terms of total viable count (TVC) and yeast/mold compared to the control and synthetic groups, respectively. This study showed that pomegranate peel extract has a better protective effect than ascorbic acid and it can be used as a natural additive in preserving the quality of seafood products.

## Graphical abstract

Sausage obtained from thornback ray (*Raja clavata*) was functionalized with pomegranate peel extract. Pomegranate peel extract added sausages gave better shelf‐life values than the synthetic antioxidant (ascorbic acid) added sausage. Pomegranate peel extract extended the shelf life of sausages by 7 more days.

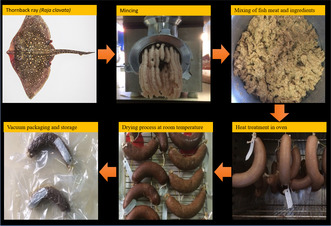

## 1 INTRODUCTION

Seafood is an easily digestible protein source containing unsaturated fatty acids with high nutritional value, which plays an important role in human nutrition (Mahdavi & Ariaii, 2021; Öğretmen, 2022). Fish contain most of the nutrients required for human nutrition (Karsli et al., 2019). Like other fish, thornback ray is rich in nutritional content, such as carbohydrates, proteins, fats, minerals, and vitamins. However, thornback ray is damaged chemically and microbiologically if it is not kept under appropriate conditions immediately after being caught. Therefore, it is necessary to use various modern and traditional processing methods to extend the shelf life of fish (Suprapto et al., 2021).

In recent years, people's changing lifestyles have led to an increase in demand ready for consumption. The increase in consumers looking for ready‐to‐eat foods has led to increasing concerns in terms of safe, quality, and healthy nutrition. Emulsified meat products such as sausage contain high levels of saturated fatty acids and cholesterol, which can threaten consumers’ health. Formulating low‐fat products with a high nutrient profile is one of the major challenges in meat processing (Pourashouri et al., 2020). For this reason, increasing the quality of emulsion meat products such as sausage has become an important problem. Therefore, sausage production from fish with high nutritional content offers great potential for the food industry (Lago et al., 2019). The use of fish mince as a raw material in ready‐to‐eat foods has recently become widespread because it is healthier, guaranteed, and cheaper (Pourashouri et al., 2020). Fish sausage products are made in the form of cylinders with a chewy texture after adding various spices and fillers to the minced meat and tend to spoil easily during storage (Rofikoh et al., 2021). It is a healthier alternative to traditional meat sausages due to its high content of unsaturated fatty acids (Feng et al., 2020). However, even in the chilled conditions of fish sausages, hydrolytic rancidity and oxidative rancidity can develop rapidly, leading to quality loss (Zakaria & Sarbon, 2018).

Natural and synthetic food additives with antioxidant effects are widely used in the meat industry to prevent the development of oxidative reactions and extend the shelf life of meat products. In parallel with this trend and the food industry's demand for naturally occurring antioxidants, natural additives are being investigated in more detail (Ahmed & Abd Elhameed, 2024; Lavado & Cava, 2023). For decades, natural antioxidants derived from fruits, vegetables, or spices have been tested and evaluated as alternatives to traditional antioxidants. These antioxidants are often added as extracts to meat products and have different antimicrobial and antioxidant activities depending on their origin (Delgado‐Pando et al., 2021). Recent studies have shown that fruits, in addition to being good antioxidants, may also be effective in preventing cancer. In parallel with the demand for naturally occurring antioxidants by consumers, researchers, and the food industry, the application of pomegranate and its byproducts has been used as a source of natural antioxidants to control oxidative processes in various meat products. Researchers observed that pomegranate and its byproducts caused a significant decrease in protein oxidation and lipid peroxidation in meat and meat products (cooked sausages, frankfurters, meatballs, steaks, nuggets, marinated meat, and hamburgers) during processing and storage (Gullón et al., 2020; Kaderides et al., 2021; Kandylis & Kokkinomagoulos, 2020; Lavado & Cava, 2023; Munekata et al., 2020; Saleh et al., 2017; Shahamirian et al., 2019; Smaoui et al., 2019). Pomegranate fruit and its byproducts contain high levels of biomolecules, including phenolic acids, flavonols, anthocyanins, and hydrolysable tannins, with significant antioxidant activities (Kandylis & Kokkinomagoulos, 2020). Approximately 54% of the pomegranate fruit consists of waste components, such as pomegranate peel (43%) and seeds (11%) (Ko et al., 2021), and it is very important to benefit from the important biological activities of these waste products such as antioxidant and antimicrobial.

The thornback ray, which is generally obtained as a nontarget hunting product in Türkiye, does not have a commercial catch and does not see a demand for consumption. Sausage, which is one of the traditional tastes and aromas of Türkiye's meat products, is filled into artificial or natural casings by mixing meat and oil with various additives (salt, sugar, garlic, spices, etc.) after passing through the meat grinder. It is a long‐lasting fermented product obtained by ripening under certain temperatures, humidity, and air speed conditions (Kara & Akkaya, 2010). Food safety has a direct impact on human health and quality of life. Therefore, it is a serious concern for both the consumer and the food industry. In this context, the aim of this study is (1) to determine the shelf life of the sausage obtained from stingray fish, which is defined as discarded fish and not consumed in Türkiye, by monitoring and evaluating its storage in refrigerator conditions, and (2) to investigate the protective effect of pomegranate peel extract added to sausage dough against ascorbic acid, one of the synthetic antioxidants.

## 2 MATERIALS AND METHODS

### 2.1 Raw material

Stingrays were captured in southeastern Black Sea in 2018–2019. A total of 29 stingray fish were used in the study. The individuals ranged from 68 to 89 cm in total length, 46 to 56 cm in disk width, and 1.07–3.63 kg in disk weight. The caught fish were frozen in a Styrofoam box and transferred to the laboratory of the Fish Processing Technology. The samples were kept in a cold room at −20°C until sausage production.

### 2.2 Sausage process

The skins of the cut wings were peeled, and the meat parts were obtained and passed through the meat grinder together with the tail fat to bring them to the form of fish minced meat. To completely drain the water of the chopped fish, it was kept in the refrigerator at +4°C overnight. At the same time, spices, sausage cases, and other additives to be used in sausage production were autoclaved at 121.1°C for 15 min. The ingredients used in the sausage dough and the mixture ratios are indicated in Table 1.

**TABLE 1:** Proportions of raw materials and additives used in the production of fish sausage.

Ingredients	Sausage mixture ratios (%)
Fish meat	85
Antioxidant	5
Salt	1.5
Red pepper	1
Black pepper	0.25
Cumin	0.25
Ginger	0.25
Allspice	0.25
Sugar	0.25
Garlic	1
Coriander	0.25
Beef tallow	2
Wheat starch	3

**FIGURE 1:**
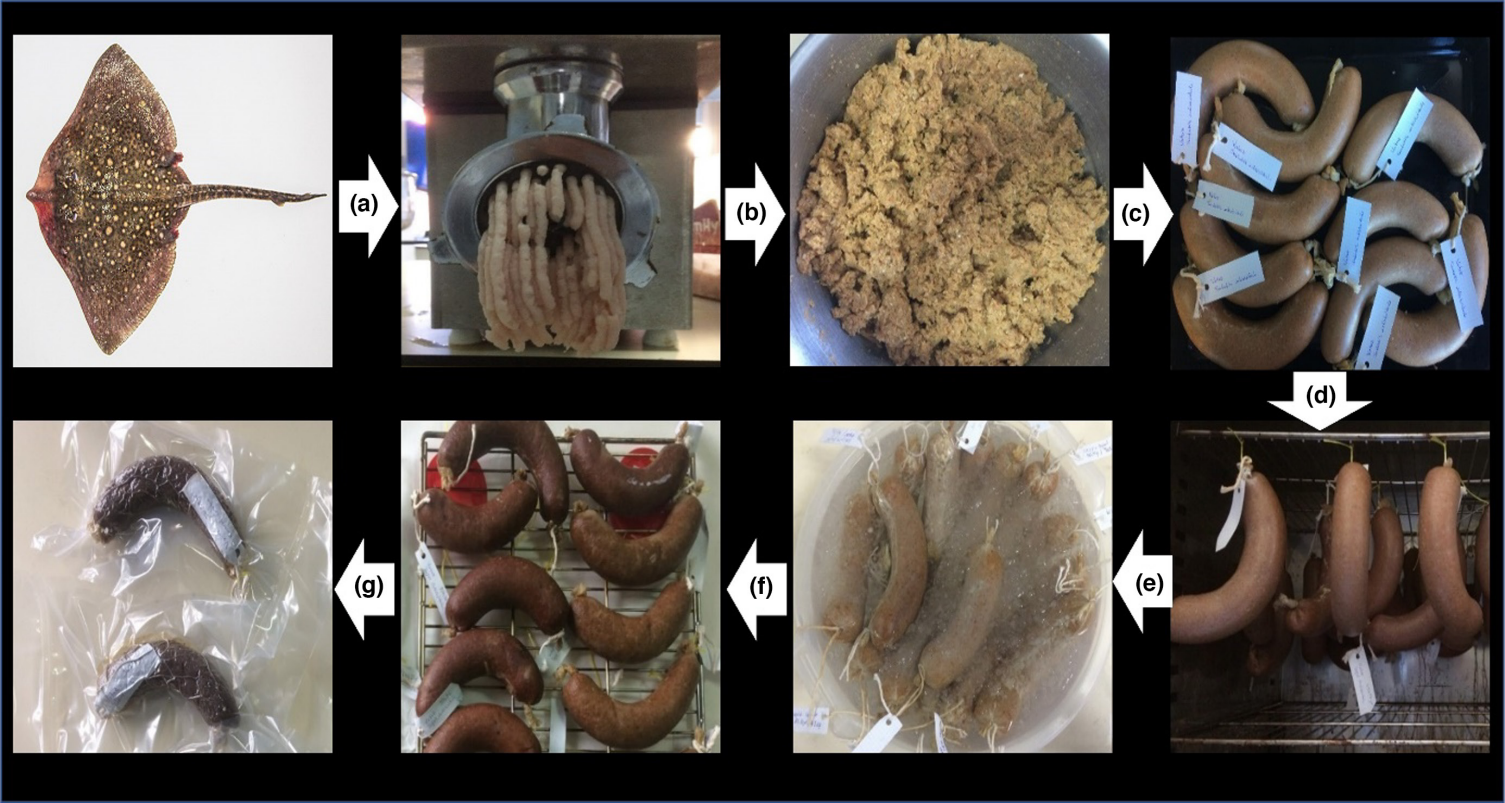
Flow diagram of sausage production. (a) mincing, (b) mixing of fish meat and ingredients, (c) stuffed sausage, (d) heat treatment in oven, (e) cooling process in ice water, (f) drying process in room, and (g) vacuum‐packaged final product.

The sausage ingredients prepared according to the above formulation were mixed for 15 min with the help of a dough‐kneading machine to obtain a homogeneous mixture. After the resulting mixture is kept for an hour under refrigerator conditions, it is filled into natural intestinal covers. Fish sausages were prepared into three different groups (Control group: antioxidant non‐added, natural group: pomegranate extract added, and synthetic group: ascorbic acid added group). The prepared fish sausages were hung in the drying oven so that they do not touch each other, and heat treatment was applied at 85°C for 45 min. After the heat treatment application, the sausages were cooled for 15 min in water:ice mixture prepared in a 1:1 ratio. After the cooling process, the sausages were left to the natural drying process in outdoor conditions so that they do not receive direct sun. After the drying process, the sausages were packaged with vacuum packaging technology using vacuum bags of 20 cm width and 34 cm length and stored at +4 ± 1°C. The workflow chart for sausage production is given in detail in Figure 1.

### 2.3 Proximate analyses

The proximal composition of the fish sausages was determined according to the methodology described by the Association of Official Analytical Chemists. According to this methodology, moisture content (AOAC, 1995, Method 985.14) was determined in an oven at 105°C, and ash content (AOAC, 1980, Method 7.009) was determined by gravimetric method by burning at 550°C. The protein content (AOAC, 1980, Method 2.507) was determined according to the micro‐Kjeldahl method using a 6.25 nitrogen conversion factor. Lipid content (AOAC, 1990) was detected by a Soxhlet extractor device (Velp SER 148/6, Milano, Italy) with petroleum ether. Carbohydrates were calculated as follows:

$$\text{Carbohydrate}\;\left(\%\right)=\left[100–\text{moisture}\;\left(\%\right)-\text{crude}\;\mathrm{fat}\;\left(\%\right)-\text{crude protein}\;\left(\%\right)-\mathrm{ash}\;\left(\%\right)\right].$$

### 2.4 Physicochemical analyses

Water activity (aw) analysis was performed in specific equipment (Aqua Lab 4TE, USA). The pH of the product was measured using a digital pH meter (Hanna, HI 3220, Hanna Instruments, Germany) with an immersion electrode. The color analysis was performed in a colorimeter instrument (Konica Minolta CR‐10, Japan), defining the chromatic space in rectangular coordinates (L*, a*, b*). The level of total volatile basic nitrogen (TVB‐N) was determined following the distillation procedure reported by the Lücke‐Geidel method (Varlık et al., 1993). Thiobarbituric acid (TBA) level was determined according to the method proposed by Tarladgis et al. (1960) and oxidation products were quantified as malondialdehyde (mg of MDA/kg) equivalents.

### 2.5 Microbiological analyses

For microbiological analysis, 25 g of the sample was transferred to a stomacher bag, and then 225 mL of sterile 0.1% peptone water was added and homogenized in a stomacher device (Interscience‐BagMixer 400, Interscience, St Nom, France). Thus, the sample was diluted to 10^−1^. This dilution was achieved up to 10^−6^ using the same diluent (Halkman, 2005). Samples from serial dilutions (0.1 mL) of stingray homogenates were spread on the surface of the agar plates. The total viable bacteria count was determined using the Plate Count Agar (Merck, Germany) and plates were incubated at 37°C for 24 h. Potato Dextrose Agar medium (Merck, Germany) was used for counting yeast–mold in the samples and incubated at 25°C for 5 days. Microbial counts were presented as logarithm colony‐forming units (log CFU)/g sample (ICMSF, 1986).

### 2.6 Statistical analysis

The mean ± standard deviation (n:2–3) of the data obtained in the study was given. Means were statistically compared by one‐way analysis of variance (ANOVA). When ANOVA determined significant differences (*p* < .05) between the mean values, the smallest significant difference (least significant difference (LSD)) test was used to assess the significance of the differences between pairs of group means. Statistical analyses were performed using JMP 5.0.1. SAS (SAS Institute Inc, NC, USA) software.

## 3 RESULTS AND DISCUSSION

### 3.1 Biochemical composition

The proximate composition of fresh and sausage thornback ray is given in Table 2. The amount of crude protein in fresh thornback ray was found as 20.43%. The amount of protein was determined as 20.16% in the control group, 20.43% in the synthetic group, and 21.12% in the natural antioxidant group on the 0th day of storage (*p* > .05). Due to the heat treatment and drying processes, there was a loss of water in the sausages over time, and accordingly, a proportional increase in the amount of dry matter occurred. Therefore, proportional increases were observed in the amount of crude protein depending on the increased dry matter content. At the end of the storage period, the protein values of the control, synthetic, and natural groups were determined as 42.91%, 43.40%, and 42.67%, respectively (*p* > .05). No significant differences were observed among the groups during the storage time (*p* > .05); however, the protein content increased significantly from the 15th day of refrigerated storage (*p* < .05). The amount of fat in fresh thornback ray was 0.39% and this rate was determined as 1.58% in the control group, 2.19% in the synthetic group, and 2.06% in the natural antioxidant group on the 0th day of storage (Table 2). On the 15th and 21st days of storage, fat values of sausages were found significantly higher than those of the 1st day and fresh thornback ray (*p* < .05). It was observed that the additives and drying process were effective in fat changes. On the 15th day, the highest fat content was found to be 4.93% in the natural group. The amount of moisture in fresh thornback ray was determined as 76.78%. The moisture contents of the control, synthetic, and natural sausage groups were found to be 69.27%, 68.65%, and 67.44% on day 0, respectively (Table 2). Depending on the addition of additives and drying process in the sausage production steps, the moisture content of samples significantly increased in all three sausage groups on the 15th and 21st days of storage and the differences observed were considered statistically significant (*p* < .05). The crude ash value of fresh thornback ray was found to be 1.83% (Table 2). Ash contents of sausages on the 0th day of storage were determined as 3.39% in the control group, 3.43% in the synthetic group, and 3.48% in the natural antioxidant group. The crude ash content increased from the 15th day, and this value was found to be statistically higher on the 15th and 21st days of storage compared to the results of fresh and 0th day (*p* < .05). The highest crude ash content was detected as 7% in the synthetic group on the 15th day. The carbohydrate content was determined as 0.57% in raw thornback ray, while this value increased significantly in all sausage groups (*p* < .05). The addition of ingredients contributed to the carbohydrate contents.

**TABLE 2:** Changes in nutritional composition (%) of thornback ray sausages during refrigerated storage.

Groups	Storage days	Crude protein	Crude fat	Moisture	Crude ash	Carbohydrate
Fresh		20.43 ± 0.12_A_	0.39 ± 0.07_C_	76.78 ± 0.02_C_	1.83 ± 0.07_C_	0.57 ± 0.01_D_
Control	0	20.16 ± 0.00^a^_A_	1.58 ± 0.60^a^_A_	69.27 ± 0.04^a^_A_	3.39 ± 0,08^a^_A_	5.60 ± 0.09^a^_C_
	15	39.87 ± 1.49^b^_B_	4.38 ± 0.14^b^_B_	42.88 ± 0.73^b^_B_	6.63 ± 0.17^b^_B_	6.24 ± 0.07^b^_B_
	21	42.91 ± 1.69^b^_B_	3.98 ± 0.09^b^_B_	41.12 ± 0.52^b^_B_	6.42 ± 0.01^b^_B_	5.57 ± 0.12^a^_C_
Synthetic	0	20.43 ± 0.10^a^_A_	2.19 ± 0.07^a^_A_	68.65 ± 0,00^a^_A_	3.43 ± 0.05^a^_A_	5.30 ± 0.08^a^_C_
	15	44.15 ± 0.31^b^_B_	4.46 ± 0.14^b^_B_	38.56 ± 0.07^b^_B_	7.00 ± 0.09^b^_B_	5.83 ± 0.15^b^_B_
	21	43.40 ± 0.67^b^_B_	4.32 ± 0.01^b^_B_	38.92 ± 0.35^b^_B_	6.82 ± 0.1^b^_B_	6.54 ± 0.18^c^_A_
Natural	0	21.12 ± 0.04^a^_A_	2.06 ± 0,06^a^_A_	67.44 ± 1.09^a^_A_	3.48 ± 0,07^a^_A_	5.90 ± 0.06^a^_B_
	15	40.60 ± 1.32^b^_B_	4.93 ± 0.36^b^_B_	42.64 ± 0.07^b^_B_	5.92 ± 0.01^b^_B_	5.91 ± 0.04^a^_B_
	21	42.67 ± 0.28^b^_B_	3.69 ± 0.23^b^_B_	41.94 ± 0.81^b^_B_	6.38 ± 0.04^b^_B_	5.32 ± 0.11^b^_C_

*Note*: Different lowercase letters (a–‐c) in the same column represent statistical difference within the same group on different days (*p* < .05). Different capital letters (A–D) in the same column represent statistical difference among different groups within the same day (*p* < .05).

The principal composition of fish varies between 66%–84% water, 15%–24% protein, 0.1%–22% fat, 0.8%–2% mineral substance, and 0–0.5% carbohydrate (Karslı & Çağlak, 2021; Love, 1970). Fish contains a very small amount of carbohydrates. The biochemical composition of seafood varies from species to species, and even among individuals of the same species according to age, sex, season, hunting region, and environmental conditions (Çağlak & Karsli, 2017). According to the Turkish Standards Institute (TS‐1070), the moisture content in the sausage should be a maximum of 40% (TSI, 2012). Ayas et al. (2019) reported that the fat contents in the muscle tissue of four different ray species (*Dasyatis pastinaca*, *Raja radula*, *Raja clavata*, and *Torpedo marmorata*) were 1.62%, 1.31%, 1.20%, and 1.43%, respectively. Özer et al. (2012) stated that the moisture, protein, fat, and ash contents of vacuum‐packed sausage made by hot‐smoking from *R. clavata* were 24.92%–33.66%, 21.57%–22.35%, 0.43%–2.40%, and 1.47%–3.73%, respectively. In another study, the moisture, protein, fat, and ash contents of fresh thornback ray were 76.51%, 18.58%, 3.39%, and 1.1%, respectively (Colakoglu et al., 2011). In addition, they found that these values were 61.36%, 30.62%, 4.41%, and 2.65% in the smoked thornback ray, respectively. Yılmaz and Akpınar (2003) stated that the moisture, protein, fat, and ash contents of the common guitarfish (*Rhinobatos rhinobatos*) during cold storage ranged from 75.83% to 79.88%, 16.63% to 22.63%, 0.2% to 0.7%, and 1.00% to 1.65% during the 6‐month frozen storage period. Kahraman and Berik (2013) found that the moisture, protein, fat, and ash contents of sausage from spiny dogfish (*Squalus acanthias*) were 47.44%, 30.21%, 16.37%, and 5.38%, respectively. In a study, the moisture, protein, fat, ash, and carbohydrate contents of five different Malaysian commercial fish sausages were in the range of 67.33%–73.36%, 8.18%–10.77%, 0.93%–6.53%, 1.71%–2.61%, and 12.30%–19.59%, respectively (Shamsudin et al., 2014). In a 30‐day sausage production study from carp, they found moisture, fat, and protein values in the ranges of 38.02%–51.58%, 24.02%–30.76%, and 21.02%–29.53%, respectively, during the study period (Arslan et al., 2001a).

As a result of the studies, it was determined that the moisture, crude fat, crude protein, and crude ash values obtained were in parallel with the nutritional contents obtained in our research. In the present study, it was observed that there was a decrease in the amount of moisture and an increase in the amount of fat and protein. The loss of moisture content in thornback ray sausages during cold storage may be due to the sausage processing steps and the decrease in protein solubility and, accordingly, the decrease in the water retention capacity of the sausages (Abou‐Taleb, 2022). The proportional increase in the amount of fat and protein in sausage groups was due to this increase in moisture. Also, it is thought that the increase in the amount of ash is due to the decrease in moisture as well as the additives used in sausage making.

### 3.2 Physicochemical analyses

Total volatile basic nitrogen (TVB‐N) is commonly used as a criterion for spoilage in aquatic products, but this criterion may not apply to cartilaginous species such as thornback ray (Özer et al., 2012). Because the tissue of ray contains high amounts of urea, the high TVB‐N could be attributed to the presence of ammonia in the tissue (Boziaris, 2014). Cartilaginous fish contain higher levels of nonprotein nitrogenous compounds than other aquaculture products due to their biological structure, therefore higher TVB‐N limit values such as 50 mg/100 g and 100 mg/100 g have been reported in the literature for cartilaginous fish (Uyttendaele et al., 2018; Varlık et al., 1993). In the present study, the initial TVB‐N value of fresh thornback ray was determined as 31.08 mg/100 g, and this value reached the range of 75.32–98.60 mg/100 g on the 15th day of storage after being kept for drying at room temperature (Table 3). During storage, these values were found to be on an upward trend for all groups. On the 21st day of storage, TVB‐N values were found to be 125.19 mg/100 g, 108.84 mg/100 g, and 78.14 mg/100 g in the control, synthetic, and natural groups, respectively. It was determined that intragroup changes were important throughout the study period (*p* < .05). Considering the limit value of 100 mg/100 g during the study period, it was observed that the TVB‐N value of the control and synthetic sausage groups exceeded the limit value at the end of the storage, while the natural additive sausage group remained within the limit value during the storage period. These results were also in agreement with the total bacteria count of sausage groups and there was a significant reduction in the TVC of the natural group compared to other groups. Similarly, high initial TVB‐N values of rays were reported in thornback ray (Karsli & Caglak, 2021; Múgica et al., 2008; Özer et al., 2012). Özer et al. (2012) reported that the TVB‐N value of sausage from thornback ray showed a linear increase after the 21st day of storage and it did not exceed the limit value of 50 mg/100 g at the end of the 56‐day storage period at +4°C. Múgica et al. (2008) stated that the TVB‐N value of thornback ray kept in slurry and flake ice during cold storage will reach 100 mg/100 g in 4.5 days and 6.3 days, respectively. In another study, the TVB‐N values of fresh and sausage products from spiny dogfish were 13.52 mg/100 g and 34.80 mg/100 g, respectively (Kahraman, 2010). Similarly, Çağlak et al. (2022) reported that the increase in TVB‐N value of trout sausages containing pomegranate peel extract was less than the control and synthetic additive groups. Hossain et al. (2023) reported that pomegranate peel waste extract significantly reduced the increase in the TVB‐N value of Ompok pabda fish. The low amount of TVB‐N in the natural group indicates the significant positive effect of the addition of pomegranate peel extract on the inhibition of microbial growth, especially proteolytic microorganisms that cause protein degradation and give rise to volatile nitrogen compounds (Abou‐Taleb).

**TABLE 3:** Physicochemical changes in thornback ray sausages during refrigerated storage.

Groups	Storage days	TVB‐N (mg/100 g)	TBA (mg MA/kg)	pH	a_w_	Color		
						L*	a*	b*
Fresh		31.08 ± 0.00_E_	0.17 ± 0.02_C_	6.63 ± 0.12_A_	0.987 ± 0.004_A_	54.90 ± 0.70_A_	5.55 ± 0.49_C_	12.60 ± 0.28_D_
Control	0	29.56 ± 3.98^a^_E_	0.87 ± 0.03^a^_A_	6.44 ± 0.01^a^_A_	0.976 ± 0.004^a^_A_	41.05 ± 0.63^a^_C_	11.47 ± 0.04^a^_A_	22.65 ± 0.49^a^_A_
	15	98.60 ± 7.98^b^_C_	0.53 ± 0.01^b^_B_	5.96 ± 0.09^b^_B_	0.882 ± 0.064^b^_B_	45.40 ± 0.42^b^_B_	12.80 ± 0.28^b^_A_	25.80 ± 0.09^b^_A_
	21	125.19 ± 5.1^c^_A_	1.04 ± 0.01^a^_A_	5.82 ± 0.02^b^_B_	0.886 ± 0.004^b^_B_	39.30 ± 0.70^c^_C_	10.35 ± 0.21^c^_B_	23.50 ± 0.98^a^_A_
Synthetic	0	27.45 ± 0.99^a^_E_	0.58 ± 0.07^a^_B_	6.23 ± 0.11^a^_A_	0.968 ± 0.062^a^_A_	42.51 ± 0.53^a^_C_	13.02 ± 0.34^a^_A_	25.97 ± 0.81^a^_A_
	15	98.56 ± 1.48^b^_C_	0.47 ± 0.02^a^_B_	6.25 ± 0.02^a^_A_	0.882 ± 0.006^b^_B_	45.90 ± 1.27^a^_B_	13.50 ± 0.21^a^_A_	27.40 ± 0.11^b^_A_
	21	108.84 ± 1.99^c^_B_	0.96 ± 0.17^b^_A_	5.80 ± 0.01^b^_B_	0.875 ± 0.003^b^_B_	38.20 + 0.01^b^_D_	12.55 ± 0.49^a^_A_	27.25 ± 0.35^b^_A_
Natural	0	27.45 ± 2.98^a^_E_	0.44 ± 0.02^a^_B_	6.42 ± 0.1^a^_A_	0.974 ± 0.039^a^_A_	34.10 ± 0.26^a^_E_	13.40 ± 0.09^a^_A_	20.50 ± 0.13^a^_B_
	15	75.32 ± 1.96^b^_D_	0.51 ± 0.02^a^_B_	6.34 ± 0.17^a^_A_	0.896 ± 0.098^b^_B_	33.60 ± 0.42^a^_E_	12.60 ± 0.28^b^_A_	20.60 ± 0.74^a^_B_
	21	78.14 ± 0.98^b^_D_	1.03 ± 0.02^b^_A_	5.65 ± 0.16^b^_B_	0.895 ± 0.009^b^_B_	31.05 ± 0.07^b^_E_	11.55 ± 0.07^c^_A_	17.70 ± 1.05^b^_C_

*Note*: Different lowercase letters (a–c) in the same column represent statistical difference within the same group on different days (*p* < .05). Different capital letters (A–E) in the same column represent statistical difference among different groups within the same day (*p* < .05).

Lipids in seafood are more exposed to oxidation than other meats due to their high unsaturation. Due to lipid oxidation, fatty acids and peroxides are formed first, and then aldehydes and ketones are formed, which cause unpleasant odor, discoloration, and rancidity with the oxidation of peroxides. Thiobarbituric acid (TBA) is one of the indicators used to determine the degree of secondary lipid oxidation in seafood (Cheng et al., 2021; Karsli, Caglak, & Prinyawiwatkul, 2021c). The consumable TBA limit value of seafood products has been set as 8 mg malonaldehyde/kg according to Schormüller (1969). In addition, a TBA value less than 1 mg malondialdehyde/kg is considered “excellent”, 3 mg malonaldehyde/kg “very good”, and 3–5 mg malonaldehyde/kg as “good quality”. TBA value was found as 0.17 mg MDA/kg in fresh thornback ray and a slight increase was observed in all groups during the storage period (Table 3). The maximum TBA value was 1.04 mg MD/kg in control at the end of the refrigerated storage. All sausage groups remained around 1 mg malonaldehyde/kg, indicating “excellent” quality during the refrigerated storage. However, the TBA values obtained from all groups at the end of the storage period statistically differed from other days within the group (*p* < .05). The rate of lipid oxidation in seafood depends on several factors, such as the fat content of fish, storage conditions, and packaging method. The low TBA values determined in thornback ray sausage in the present study can be attributed to the low‐fat content of thornback ray and the application of vacuum packaging. TBA values of the sausage in the present study were consistent with the literature data. Similarly, low TBA values were reported in surimi from thornback ray at the end of the 6‐month storage by Turan and Sönmez (2007), in vacuum‐packed sausage from thornback ray at the end of the 56‐day chilled storage by Özer et al. (2012), in shark sausages by Kahraman (2010), and in rohu fish sausage stored at room and 5°C by Sini et al. (2008). In addition, the effect of pomegranate peel extracts on reducing lipid oxidation in uncured sausages (Lavado & Cava, 2023), Iberian dry uncured sausages (Cava & Ladero, 2023), Tuscan sausages (Zago et al., 2020), and fish meat (Çağlak et al., 2022; Dogra & Gandotra, 2024; Sabeeh et al., 2021) has also been reported. The antioxidant activity of polyphenols in pomegranate is associated with their properties as scavengers of ROS and reactive nitrogen species (RNS), direct radical scavengers of peroxidation products of proteins, lipids, RNA, DNA, and chelators of metal ions (Lavado & Cava, 2023).

It is stated that the pH value for fresh fishery products is 6–6.5 and the consumability limit value is 6.8–7 (Connell, 1980; Varlık et al., 1993). According to the Turkish Standards Institution (TS‐1070), the pH of sausage production should be between 5.4 and 5.8 (TSI, 2012). In the present study, the pH of fresh thornback ray was measured as 6.63, a decrease in pH value was observed in all groups due to sausage treatment (Table 3). The pH value was found in the range of 5.82–6.44 in the control group, 5.80–6.25 in the synthetic group, and 5.65–6.42 in the natural group during the storage period. The lowest pH value (5.65) was measured in the natural group at the end of the storage period. At the end of the storage period, a decrease in the pH value of all groups was detected and this change was found to be statistically significant (*p* < .05). However, no significant difference was observed among groups on the 21st day of the storage (*p* < .05). This reduction is probably related to the moisture loss and the additives used in sausage production. Similarly, Arslan et al. (2001b) found that the pH changes of four different fermented sausage groups made from silverfish tended to decrease during the study period. Múgica et al. (2008) stated that the initial pH value of *R. Clavata* stored in slurry ice was around 6.5. Yılmaz and Akpınar (2003) found that the pH values of the frozen common guitarfish were between 6.50 in fresh and 6.82 at the end of 6 months of frozen storage. Özer et al. (2012) reported that the pH values of control and vacuum‐packed sausage from thornback ray were reported as 6.41 and 6.18, respectively. The pH values of fillet and spiny shark sausages were reported as 6.44 and 6.70, respectively (Kahraman & Berik, 2013). Arslan et al. (2001a) found that the pH value of carp sausages kept at four different temperatures for 30 days was between 5.46 and 5.8. The pH of fish sausage produced from *Capoeta umbla* ranged from 6.3 on day 0 and 6.5 at the end of the 56th‐day storage (Özpolat & Guran, 2017). The pH value determined in the fresh thornback ray in the present study was similar to the findings of the studies carried out on different ray species in the literature. Similarly, it was also reported that the pH value of Tuscon sausages (Zago et al., 2020) and trout sausages (Çağlak et al., 2022) treated with pomegranate extracts ranged from 5.66 to 6.16 and from 6.09 to 6.20, respectively.

Water is one of the most important factors driving the spoilage reaction in foods. In particular, the course of microbial growth or degradation is closely related to the free or bound water content of food (Certel & Ertugay, 1996). It is stated that when the meat is processed into sausage dough, the water activity decreases to about 0.96 levels (Heperkan & Sözen, 1988). In the present study, the water activity (a_w_) value of fresh thornback ray was found to be 0.987 (Table 3). On the 0th day of storage, the water activity values of the control, synthetic, and natural groups were determined as 0.976, 0.968, and 0.974, respectively. Due to the drying of the sausage products, water loss occurred in the products and accordingly, a decrease in the water activity values was determined (*p* < .05). At the end of the storage, the a_w_ values of the control (0.886), synthetic (0.875), and natural (0.895) groups were determined to be statistically higher than those of fresh and day 0 (*p* < .05). Similarly, Çağlak et al. (2022) reported that a_w_ values of trout sausages treated with ascorbic acid and pomegranate extract were 0.8921 and 0.8781 at the end of the storage day, respectively. Oliveira Filho et al. (2010) reported that the average water activity value of different sausage groups formulated with minced *Nile tilapia* fillet waste was 0.98. In a study investigating the effects of starter cultures on turkey sausages, it was stated that the initial a_w_ value decreased from 0.957 to 0.917, depending on the sausage production steps (Çiçek et al., 2014). They also stated that the water activity values of fermented turkey sausages showed slight decreases after the heating step. As in other studies, the water activity values obtained in the present study decreased depending on the sausage processing steps. Especially on the 15th day, the water activity values of all sausage groups decreased significantly (*p* < .05). However, no significant difference was observed among the water activity values of the sausage groups throughout the entire storage period (*p* > .05).

The parameters that have the first effects on the evaluation of a food for the consumer are the appearance and color of the food. For this reason, we can say that these parameters are the most important quality features for consumers in consumption and purchasing decisions (Karsli, Caglak, & Prinyawiwatkul, 2021b). According to color analysis results, the L*, a*, and b* values of fresh thornback ray were 54.90, 5.55, and 12.60, respectively. L* values of all sausage products decreased (*p* < .05) during the study period and the lowest value was observed in the natural additive sausage group (31.05). Also, significant differences (*p* < .05) were observed among groups during storage periods, except for the 21st day. It was determined that the a* values obtained from sausage during the storage period were higher than those from the fresh sample (*p* < .05). The lowest and highest a* values of the control, synthetic, and natural sausage groups were found as 10.35–12.80, 12.55–13.50, and 11.55–13.40, respectively. Similarly, the b* value, which was determined as 12.60 in fresh thornback rays, increased in sausage groups because of the applied treatments. The b* values of the natural sausage group were found to be significantly lower than those of the control and synthetic groups (*p* < .05).

Color is a visual feature that occurs as a result of the spectral distribution of light (Altuğ & Elmacı, 2005). The structure of fish meat does not show a characteristic appearance that has certain standards in terms of color like some other foods. In this respect, there was no consistent relationship among color values of all groups. The physical and chemical changes that occur due to different processing techniques and storage conditions applied to aquatic products also cause changes in the color, appearance, and texture of the products. In the present study, while the L* values of thornback ray sausage decreased due to ripening, this decrease was more in the natural group with pomegranate extract. This result is in agreement with the study data of Çağlak et al. (2022) in terms of the decrease in the L* value of trout sausages depending on the ripening stage. In the present study, the a* and b* values of fresh thornback ray increased significantly as a result of sausage processing (*p* < .05). While there was no difference among the a* values in the sausage groups (*p* > .05), the b* values of the natural group differed statistically from those of the other groups (*p* < .05). Similarly, Kahraman (2010) reported that the a* and b* values of fresh fish shark meat increased in sausages depending on the applied treatments and additives. Contrary to our study, they determined a higher L* value in sausage compared to fresh shark meat. The reductions in L* are explained by changes due to moisture losses during drying and oxidation (Kahraman, 2010). Similar to the L values determined in this study, Soyer et al. (2005) reported that the L* amount of sheep sausage is between 34 and 40. The L*, a*, and b* values of five different sausages sold in Malaysia were in the range of 58.73–79.56, 0.58–17.43, and 12.69–26.55, respectively (Shamsudin et al., 2014). In another study, L*, a*, and b* values of carp sausages prepared with green tea extract were determined to be in the ranges of 67.85–81.6, 5.9–7.5, and 9.4–14.5, respectively (Pourashouri et al., 2020). It is thought that the changes observed in the colors of sausage products are due to the applied heat treatment and additives, as well as the fact that the structure of fish meat does not have a characteristic appearance with certain standards in terms of color, like some other foods (Karsli, Caglak, & Kilic, 2021).

### 3.3 Counts of total viable bacteria (TVC) and yeast/mold

As can be seen in Table 4, the initial total viable bacteria and yeast/mold counts of thornback ray were determined as 3.98 log CFU/g and 1.90 log CFU/g, respectively. The TVC and yeast/mold count of all sausage groups in the present study gradually increased over the entire storage period. Over the entire storage period, these increases were statistically significant in all groups (*p* < .05). In the present study, the TVC of thornback sausages exceeded the maximum acceptable limit of 7 log CFU/g recommended by the International Commission on Microbiological Specifications for Foods (ICMSF, 1986) on the 15th day of storage in the control group and on the 21st day in the synthetic and natural groups. Compared with the control and synthetic groups, about 2.45 log CFU/g and 0.49 log CFU/g reduction were observed for the natural group on day 15 (*p* < .05). Özer et al. (2012) stated that the total mesophilic bacteria count in smoked thornback ray sausage during 56 days of refrigerated storage showed a linear increase from the beginning (4.09 log CFU/g), and this value reached 7.69 log CFU/g at the end of the storage. The TVC of fortified carp sausages by emulsion and gelled emulsion incorporating green tea extract were found between 2.77 and 4.10 log CFU/g at the end of the 30‐day storage period (Pourashouri et al., 2020). Dinçer et al. (2017) reported that the TVC of fish sausage produced from saithe (*Pollachius virens*) was 6.32 log CFU/g at the end of the 15‐day storage period. According to the Turkish Food Codex “Communiqué on Microbiological Criteria”, the acceptable limit value for yeast–mold for heat‐treated meat products (sausage, salami, meatball, etc.) is stated as 3 log CFU/g (Turkish Food Codex, 2010). In terms of yeast/mold values, the limit value reported according to the Turkish Food Codex exceeded this value on the 15th day of storage for the control and synthetic groups, while the natural group exceeded this value on the 21st day. Yeasts/molds are not part of the normal fish flora and can contaminate fish with water, ingredients, or equipment used (Demircioğlu & Öztürk, 2020). The most likely reason for these increases in yeast/mold is related to the ingredients used in sausage making. In a study conducted on fermented sausage production from *Chalcalburnus mossulensis*, it was stated that the yeast–mold values ranged between 2.60 and 4.79 log CFU/g (Arslan et al., 2001b). Arslan et al. (2001a) reported that yeast/mold counts of four different fermented sausages from carp (*Cyprinus carpio*) were in the range of 4.59 and 4.88 log CFU/g at the end of the 30‐day refrigerated storage. In the present study, the TVC and yeast/mold counts of the natural group with pomegranate extract were lower than those of the other groups throughout storage. This result confirms the ability of pomegranate extract against microbial growth in foods. It has been reported in various studies that the addition of pomegranate peel extract also limits microbial growth in fish (Dogra & Gandotra, 2024; Uçak & Elsheikh, 2023) and minced meat (Ahmet & Abd Elhameed, 2024).

**TABLE 4:** Counts of total viable bacteria (TVC) and yeast/mold of thornback ray sausages during refrigerated storage (log CFU/g).

Analyses	Groups	Storage days			
		Fresh	0	15	21
TVC	Control	3.98 ± 0.04_A_	5.32 ± 0.01_B_^a^	7.44 ± 0.07^a^_C_	NA
	Synthetic		4.41 ± 0.02^b^_B_	5.48 ± 0.08^b^_C_	7.54 ± 0.01^a^_D_
	Natural		4.23 ± 0.05^c^_B_	4.99 ± 0.03^c^_C_	7.24 ± 0.03^b^_D_
Yeast/mold	Control	1.90 ± 0.04_A_	2.98 ± 0.06^a^_B_	4.50 ± 0.09^a^_C_	NA
	Synthetic		2.67 ± 0.03^b^_B_	3.33 ± 0.01^b^_C_	5.92 ± 0.09^a^_D_
	Natural		2.45 ± 0.04^c^_B_	2.90 ± 0.03^c^_C_	4.83 ± 0,07^b^_D_

*Note*: Different capital letters (A–D) in the same row represent statistical difference within the same group on different days (*p* < .05). Different lowercase letters (a–c) in the same column represent statistical difference among different groups within the same day (*p* < .05). Abbreviation: NA, Not analyzed.

## 4 CONCLUSION

This study was carried out in order to make sausage from thornback ray stingray, which is considered as a bycatch, and to bring the different flavors created in this way to the economy. In addition, pomegranate peels, which are considered as waste, were used as a natural additive in the production of these products. For this purpose, the nutritional, physicochemical, and microbiological changes of the sausages (control, synthetic, and natural from thornback ray) were examined during the refrigerated storage. The extract obtained from the pomegranate peel was effective in delaying lipid oxidation and limiting microbial growth on the sausage obtained from the thornback ray. According to the results of the microbiological analysis, it was determined that the consumable limit values were reached on the 15th day in the control and synthetic groups, while these limit values were reached on the 21st day in the natural group. Thus, with the contribution of pomegranate peel extract, the shelf life of the thornback ray sausage was prolonged by 6 days compared to the control and synthetic groups.

Considering the aquaculture resources in Türkiye, it was seen that products with low economic value such as thornback ray will add value by turning them into a traditional taste of sausage. In addition, it was revealed that the shelf life of waste pomegranate peel extracts as a natural preservative and/or products obtained from them can be extended. The results highlight that pomegranate peel waste can be converted into value‐added products in food applications. It is thought that the results of this study are important in terms of meeting the food needs in the face of the increasing world population and will contribute to future studies.

## AUTHOR CONTRIBUTIONS

**Emre Caglak:** Conceptualization (equal); investigation (equal); methodology (equal); writing – review and editing (equal). **Ozen Yusuf Ogretmen:** Conceptualization (equal); data curation (equal); formal analysis (equal); investigation (equal); methodology (equal); visualization (equal); writing – original draft (equal). **Baris Karsli:** Conceptualization (equal); data curation (equal); formal analysis (equal); investigation (equal); visualization (equal); writing – review and editing (equal).

## CONFLICT OF INTEREST STATEMENT

The authors declare no potential conflict of interest.

## Data Availability

The data that support the findings of this study are available on request from the corresponding author.
